# Forensic Radiology: An Update

**DOI:** 10.5334/jbr-btr.1420

**Published:** 2017-12-16

**Authors:** Maria Antonietta Clemente, Luciana La Tegola, Maria Mattera, Giuseppe Guglielmi

**Affiliations:** 1 University of Foggia, IT

**Keywords:** Forensic radiology, Virtual autopsy, Virtopsy, Virtangio, MDCT

## Abstract

Forensic radiology is a specialized area of medical imaging using radiological techniques to assist physicians and pathologists in matters related to the law. The forensic application of diagnostic medical radiology can be applied in many fields; the prime target of evaluation is the osseous skeleton, but soft tissues and abdominal and thoracic viscera may offer key findings. The technological progress in clinical radiology provides a lot of potential tools to forensic radiology, allowing wider fields of applications in this matter.

Forensic medicine covers a heterogeneous group of various disciplines or subspecialties sharing a common interest: the application of specialized scientific and/or technical knowledge to aid in civil and criminal law. Among those disciplines, forensic radiology is a specialized area of medical imaging using radiological techniques to assist physicians and pathologists in matters related to the law [1,2,3].

The forensic application of diagnostic medical radiology can be applied in many fields: human identification (particularly in investigations of mass disasters and decomposed bodies), evaluation and documentation of injury or cause of death (accidental or non-accidental), criminal and civil litigation (fatal or non-fatal), administrative proceedings, education, research and administration.

Radiological determination of individual identity may be presumptive upon demonstration of pre-existing injuries, illness, or congenital and/or developmental peculiarities but radiological identification needs direct comparison of ante-mortem and post-mortem images of the body or its parts.

Evaluation of injury or death requires elements of detection, pattern recognition, interpretation and comparison, all based on radiologic experience with normal and abnormal findings.

The prime target of forensic radiological evaluation is the osseous skeleton, but in many cases the soft tissues and the abdominal and thoracic viscera may offer key findings. Osseous injuries are best detected and studied in the post-mortem state if the body parts can replicate the positions for projections.

Analysing the localization and the type of fracture can be determine whether the injury is accidental or inflicted, particularly when considering the age and expected level of activity of the individual. Some types of fractures, dislocations, and epiphyseal separations are common in the course of *normal* activities in certain age ranges; others are instead impossible to sustain accidentally in daily activities.

The configuration and direction of fractures in the skull offer information about the impact point and direction of impact, indicate the sequence of repetitive blows and sometimes, the shape of the object or weapon used.

Fractures of the hyoid bone or thyroid cornu usually suggests strangulation; in vehicular injuries, certain fracture/dislocations may actually suggest the velocity of impact or deceleration.

Gunshot wounds, missiles and other foreign bodies such as knives are the object of many forensic radiological examinations and their radiological evaluation may provide important information [Figures 1(a–b), 2(a–b), 3].

**Figure 1:**
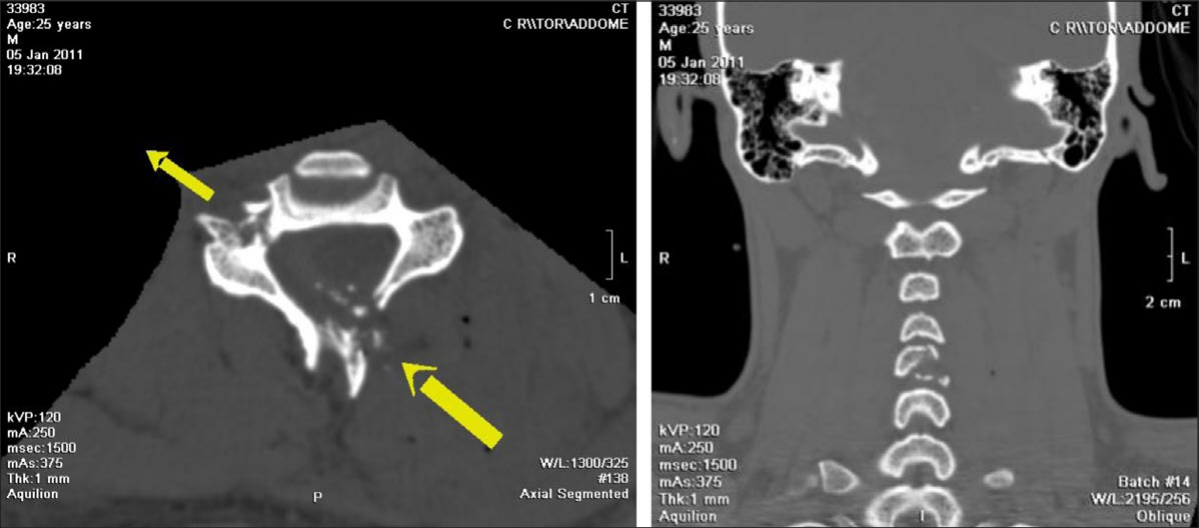
CT, axial **(a)** and coronal **(b)** sequences of the cervical spine. Fractures of the left posterior arch and of the proximal section of the C5 soma spinous process (input gunshot wound) and fracture of the right transverse C5 soma process (exit gunshot wound).

**Figure 2:**
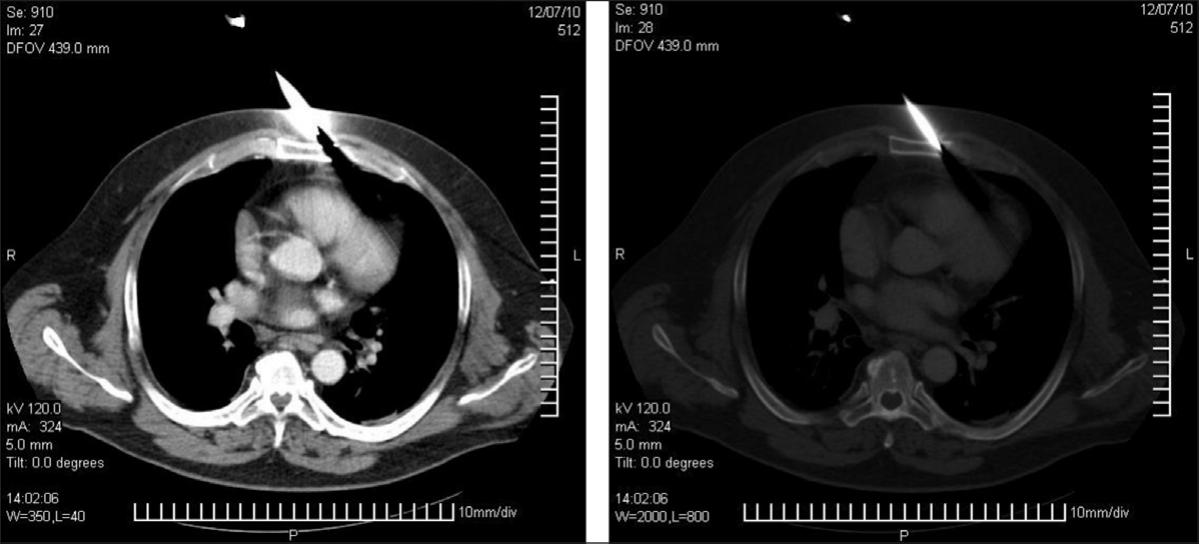
Chest CT study with contrast. A foreign metallic body (knife) causes a sternum fracture, well visible in the axial planes re-constructed with a parenchymal window **(a)** and, especially, with a bone window **(b)**.

**Figure 3:**
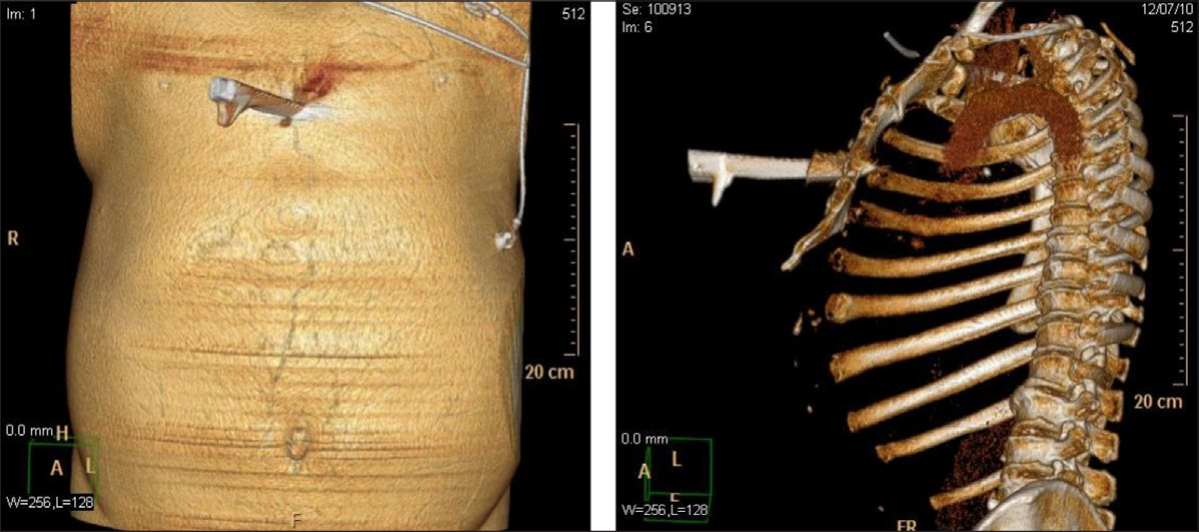
TC reconstruction. The Shaded Surface Display (SSD) highlights the blade entry point on the chest surface **(a)** and the bone penetration **(b)**.

Other trauma can include such findings as intracranial haemorrhage from shaking (battered child syndrome), penetrating wounds, which can be revealed with injected contrast media, as can vascular tears or avulsions be [4].

For all these purposes, multidisciplinary forensic teams collect data about the victims using complementary methods [5,6,7,8].

Forensic radiology requires contributions from international experts in the field of radiology and legal medicine with the aim to provide a comprehensive overview on the *state of the art* and new perspectives on future developments [9]; in fact for a long time the contribution of the radiology to the forensic medicine has been limited to the use of conventional X-ray, particularly for anthropological determination of sex, age, stature, dental status and other individual anatomic features [10].

Nowadays, with the advent of Multi Detector Computed Tomography (MDCT), the imaging spectrum has been widened because this technique provides a full-body multiplanar study which can be enhanced by several advanced image post-processing techniques [9,11,12].

MDCT plays a key-role in the detection of bone structure in trauma or of internal haemorrhage in patients with penetrating wounds allowing the identification of the entity of applied force or of the wound track, its extension, its direction and other related legal aspects [13].

These features can be very challenging for the forensic radiologist, who is supposed to have a comprehensive knowledge of the different patterns of injuries.

Despite the usefulness *in vivo*, the better application of the MDCT is in the post-mortem imaging due to its three-dimensional and multi-parametrical acquisition of the state of the cadaver before the autopsy that irreversibly changes the anatomical structures [14,15]. Unenhanced post-mortem MDCT (PMCT), however, has limitations overall in the study of vascular system. In fact, with the exception of major vascular lesions in the vascular-related pathologies, such as coronary heart disease, pulmonary embolism and others, the PMCT has demonstrated limited diagnostic value. This has led to the development of other minimally invasive techniques to perform Multiphase Post-mortem CT-angiography (MPMCTA) which link the performance of a native CT scan and three angiographic phase (arterial, venous and dynamic); thus, allowing the vascular system to be imaged in a similar way to standard clinical CT angiography [16,17].

In this technique, a mix of paraffin oil and a specially created oily contrast agent is injected via a device which reproduces the conditions of perfusion in a living body, enabling the radiologist to capture highly accurate images of any abnormalities in the vascular bed of the viscera, or lesions of the vascular system. In fact, nowadays this technique is routinely used because of better results in comparison with conventional autopsy, particularly in detecting the source of a haemorrhage and coronary occlusions [18,19].

More recently, MRI has been used to increase forensic investigations, particularly in musculoskeletal, cardiovascular and angiographic fields and in forensic imaging of the living (such as cases of child abuse), survived strangulation and age estimation [20].

Virtual Autopsy is one new technique that offers several advantages over the traditional approach and helps connect radiology with forensic medicine. The most important advantage is its non-invasive approach that doesn’t harm the body or tamper with forensic evidence [21].

The method creates 3-D models that can be easily accessed and the data quickly relayed via computer. It is currently standard procedure for forensic investigations and an emerging procedure around the world. Virtual Autopsy includes a wide range of technologies such as photogrammetry and 3-D surface scanning for the exterior and CT, MR imaging, angiography (Virtangio) and biopsy for the interior. The information produced by the individual modalities is then merged into a robotic system called Virtobot which creates 3-D, high resolution computer images to document an injury [22].

Another advantage of virtual autopsy over the conventional method is that it speeds up the decision-making process because imaging can be done so quickly. The process is also observer-independent, allowing for objective data archiving. Finally, virtual autopsies can be used in cultures and situations where conventional autopsy is not tolerated for religious reasons or is rejected by family members.

The technological progress in clinical radiology provides a lot of potential tools for forensic radiology, allowing wider fields of applications in this matter. However because it is an emerging subspecialty, it needs intensive research, standardization, guide-lines and specific training to form a better understanding of the *forensic radiologist*, which integrates the knowledge of different available imaging techniques, their strengths and weaknesses as well as their medical legal features.
